# Persistent Erythrocytosis in a Dog With a Spinal Sclerosing Paraganglioma

**DOI:** 10.1111/vcp.70112

**Published:** 2026-05-03

**Authors:** Samuel V. Neal, Samantha L. Sommer, Sarah Golconda, Eric T. Hostnik, Russell Sakacs, Janice Park, Ashley Potts, Ronaldo C. da Costa, Christopher Premanandan, Kara Corps, Sanggu Kim, Maxey L. Wellman

**Affiliations:** ^1^ Department of Veterinary Biosciences, College of Veterinary Medicine The Ohio State University Columbus Ohio USA; ^2^ Department of Veterinary Clinical Sciences, College of Veterinary Medicine The Ohio State University Columbus Ohio USA

**Keywords:** canine, erythropoietin, intradural, neoplasia, polycythemia

## Abstract

A 7‐year‐old, male, castrated, mixed‐breed dog presented with right pelvic limb lameness and pain on posturing to defecate that began 1.5 years prior to presentation. Radiographs taken 8 months prior to presentation showed a small lytic lesion of the L6 vertebral body, and CBCs showed a persistently increased hematocrit (HCT) for at least 2 years. Lumbar magnetic resonance imaging (MRI) revealed a right‐sided L5–L7 intradural‐extramedullary mass. Cytologic evaluation of a fine‐needle aspirate of the mass was consistent with a diagnosis of malignant neoplasia, with a primary differential of paraganglioma. Additional testing revealed an increased normetanephrine‐to‐creatinine ratio and low serum erythropoietin (EPO). Histologic evaluation, immunohistochemistry, and electron microscopy were consistent with a diagnosis of spinal sclerosing paraganglioma. No mutations were found on DNA sequencing that are associated with polycythemia vera in humans, a dog with primary erythrocytosis, or in humans with polycythemia–paraganglioma syndrome (PPS). The patient was treated with palliative radiation, after which the neurologic deficits markedly improved and the erythrocytosis resolved. The patient continued to do well with an HCT within the reference interval. Two years later, the patient's neurologic signs recurred, and MRI revealed the paraganglioma had progressed in size. The patient continued to have an HCT within the reference interval and underwent repeat palliative radiation with no complications. The concurrent erythrocytosis and paraganglioma in this patient resemble reports of PPS in humans.

## 1 Case Presentation

A 7‐year‐old, male, castrated, mixed‐breed dog presented for evaluation of right pelvic limb lameness and pain on posturing to defecate that began 1.5 years prior to presentation. Radiographs from the referring veterinarian 8 months prior to presentation showed a small lytic lesion of the L6 vertebral body. Repeat radiographs 4 months later showed progressive lysis encompassing both the L6 and L7 vertebrae, accompanied by worsening right pelvic limb lameness. CBCs showed a persistently increased hematocrit (HCT) for at least 2 years duration.

On physical examination, the dog was occasionally non‐weight‐bearing on the right pelvic limb, which was accompanied by severe muscle atrophy of that limb, and there was intermittent urinary incontinence. Neurologic examination revealed monoparesis of the right pelvic limb, with markedly decreased flexor reflex, marked muscle atrophy, and mild caudal lumbar pain. The lesion was localized to the L6–S3 radiculomyelopathic region with right lateralization. A CBC (ADVIA 2120i, Siemens Healthineers) showed marked erythrocytosis (HCT 78%, reference interval (RI), 40%–59%) and was otherwise unremarkable. A routine chemistry panel (Cobas c501, Roche Diagnostics) showed a moderately increased total calcium (13.3 mg/dL, RI 9.1–11.5 mg/dL). Given the presence of vertebral lysis, localized osteolysis‐associated release of calcium was considered the primary differential for the elevated total calcium, with humoral hypercalcemia of malignancy considered less likely. An ionized calcium (Nova VET Plus Prime analyzer, Nova Biomedical Corporation) was within the reference interval (5.0 mg/dL, RI 4.6–5.4 mg/dL). Total calcium was within the reference interval (10.5 mg/dL) on the next chemistry panel performed approximately 5 months after radiation therapy (discussed below). Thoracic radiographs were unremarkable. Abdominal ultrasound showed a soft tissue mass associated with a caudal lumbar vertebral body that was suspected to be L6. Lumbar magnetic resonance imaging (MRI) revealed a large (5.0 × 2.1 × 3.3 cm) multilobular, T2‐weighted heterogeneous hyperintense, T1‐weighted isointense, strongly contrast‐enhancing intradural‐extramedullary mass centered on the right lateral aspect of the L5–6 vertebral canal (Figure 1). There was extensive, expansile lysis of the vertebral bodies, pedicles, laminae, L7 spinous process, and articular processes between the midbody of L5 and the caudal endplate of L7, which was worse on the right side. The mass severely expanded the vertebral canal with leftward compression of the spinal cord and cauda equina.

**FIGURE 1:**
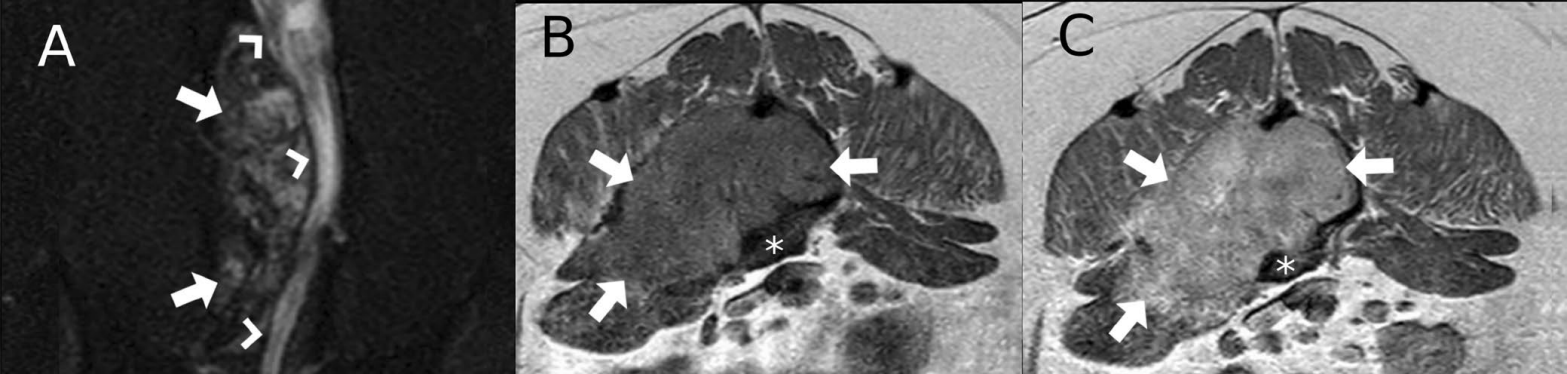
Lumbar magnetic resonance imaging (MRI). (A) Dorsal plane T2‐weighted DIXON fat suppression sequence of a right‐sided heterogeneous hyperintense mass (arrows). The mass resulted in a leftward deviation of the caudal lumbar epidural space (chevrons). (B, C) Transverse plane T1‐weighted sequence precontrast (B) and postcontrast (C). The mass (arrows) was diffusely contrast‐enhancing and resulted in expansile lysis of the vertebra (asterisks).

Cytologic examination of a fine‐needle aspirate of this mass showed frequent moderately sized, pleomorphic cells with intermediate nuclear‐to‐cytoplasmic (N:C) ratios (Figure 2). Cells often formed rosette‐like arrangements that surrounded amorphous, pink, wispy, fibrillar, extracellular material and were rarely found in large aggregates associated with vascular elements. Cells had moderate amounts of medium‐blue cytoplasm that typically contained many small, clear, discrete vacuoles. Nuclei were pleomorphic, with finely stippled chromatin and one to several small, round, prominent nucleoli. Cells displayed moderate anisocytosis and anisokaryosis, and rare binucleation with nuclear molding. The cytologic diagnosis was malignant neoplasia, and, given the location and cytomorphology, differential diagnoses included paraganglioma, ependymoma, primitive neuroectodermal tumor, and nephroblastoma.

**FIGURE 2:**
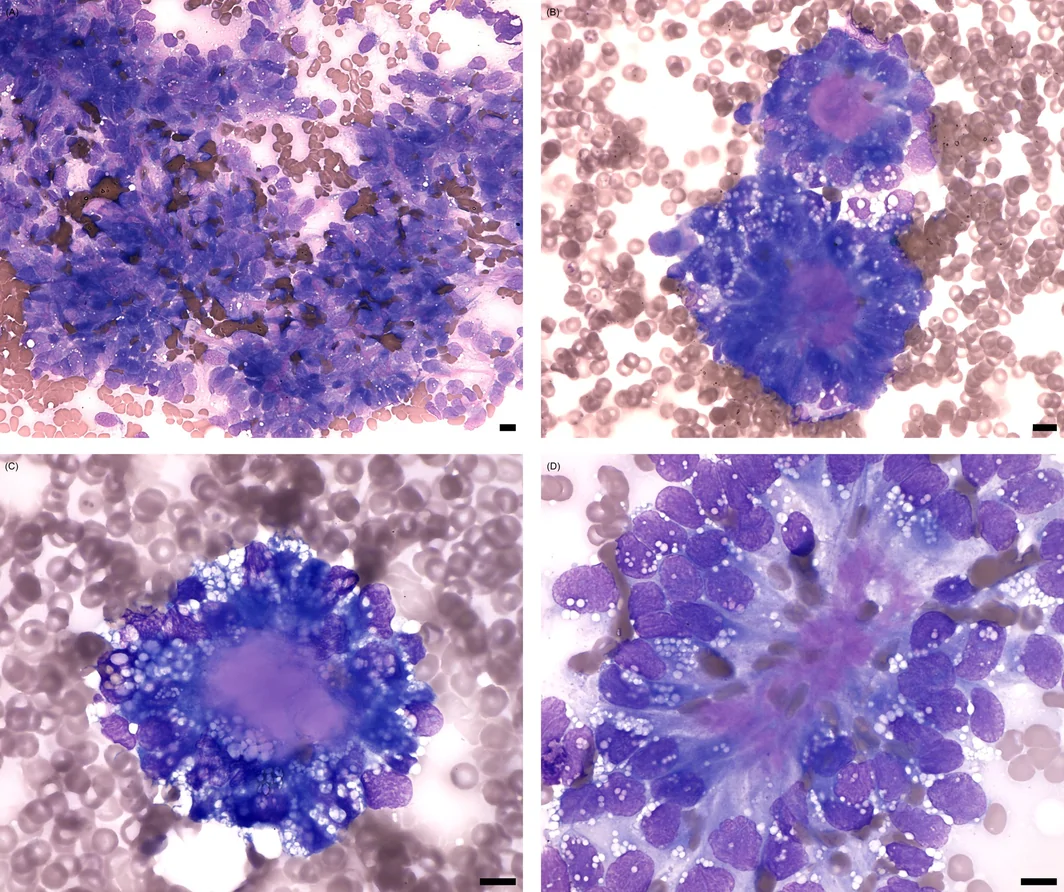
Cytology of the spinal mass. (A) Neoplastic cells were rarely found in large aggregates associated with vascular elements. Wright–Giemsa Stain, ×40 objective. (B–D) More commonly, neoplastic cells were found in rosette‐like arrangements that surrounded amorphous pink material. Wright–Giemsa Stain, (B) ×60 objective and (C, D) ×100 objective. (A–D) scale bar = 10 mm.

Based on a differential diagnosis of paraganglioma and the persistent erythrocytosis, additional laboratory testing was performed and results included an increased urine normetanephrine‐to‐creatinine ratio (855, RI 28–380, IDEXX Laboratories), a urine metanephrine‐to‐creatinine ratio within the reference interval (248, RI 18‐359, IDEXX Laboratories), and a decreased serum erythropoietin concentration (EPO) (6 U/mL, RI 7–37 U/mL, enzyme‐linked immunosorbent assay, Laboklin, Germany).

Histologic evaluation of an H&E‐stained section of a Tru‐Cut biopsy of the mass revealed an encapsulated, moderately cellular neoplasm comprising neoplastic cells arranged in haphazard short streams and bundles amongst dense collagenous stroma (Figure 3). Within areas of hemorrhage, there were rosette‐type structures consisting of similar cells surrounding foci of eosinophilic smudgy material. Neoplastic cells were polygonal to spindle‐shaped, had variably distinct cell borders, moderate amounts of eosinophilic cytoplasm, and a single oval nucleus with finely stippled chromatin and a single prominent nucleolus. Anisocytosis and anisokaryosis were moderate. Mitotic figures were rare, approximately 1 in ten 400× fields (area: 2.37 mm^2^). The histologic diagnosis was spinal sclerosing paraganglioma. Immunohistochemically, neoplastic cells diffusely exhibited positive cytoplasmic staining for neuron‐specific enolase (NSE) and stained negatively for synaptophysin, chromogranin A (CgA), and pancytokeratin (Figure 4).

**FIGURE 3:**
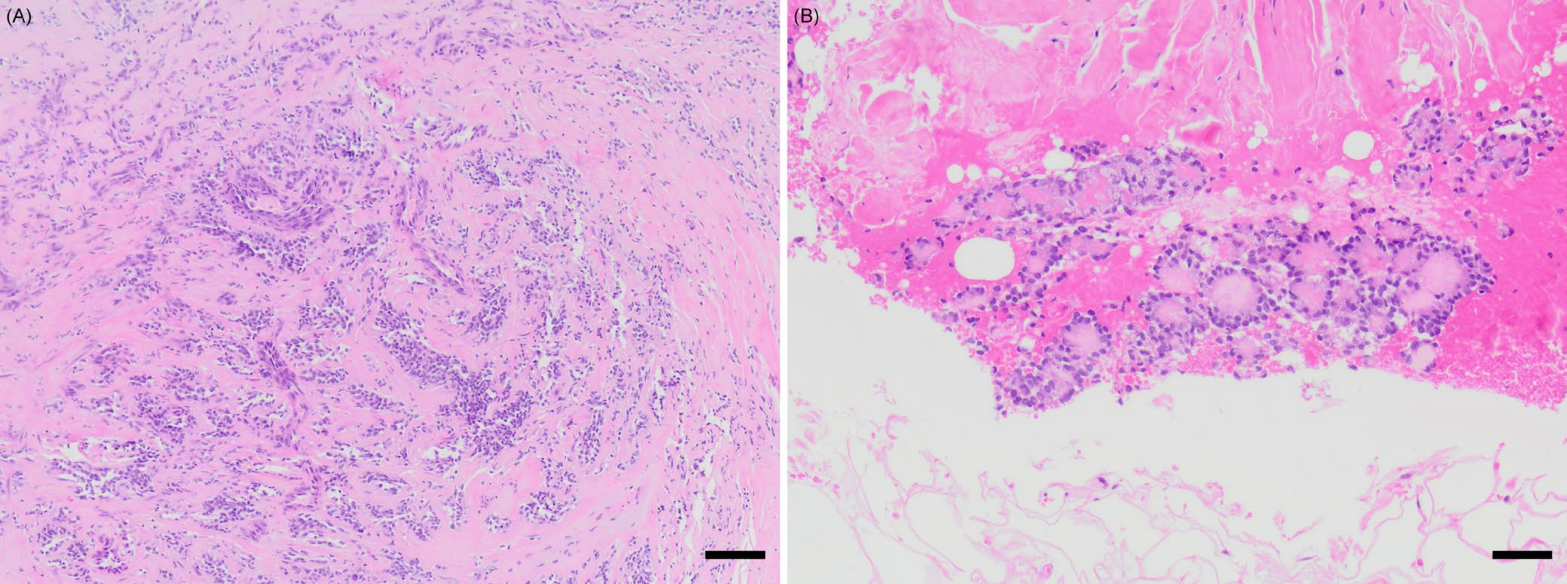
Histopathology of the spinal mass obtained through a Tru‐Cut biopsy. (A) Neoplastic cells were arranged in haphazard short streams and bundles amongst a dense collagenous stroma. H&E stain, ×10 objective. (B) Neoplastic cells were also found in areas of hemorrhage, arranged in rosette‐type structures surrounding foci of eosinophilic material. H&E stain, ×20 objective. (A) scale bar = 100 mm. (B) scale bar = 50 mm.

**FIGURE 4:**
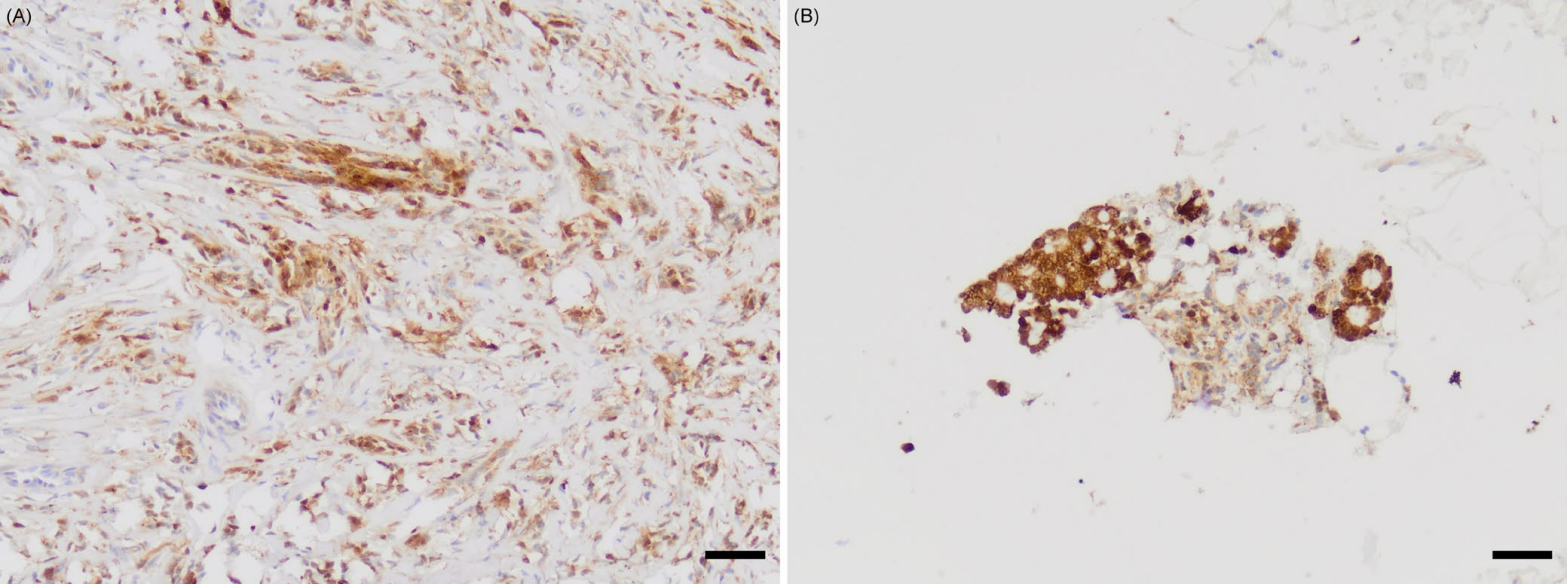
Immunohistochemistry of the spinal mass obtained through a Tru‐Cut biopsy. (A, B) Neoplastic cells displayed diffuse cytoplasmic staining for neuron‐specific enolase (NSE), ×20 objective. (A, B) scale bar = 50 mm.

Ultrastructural pathology was evaluated by transmission electron microscopy, revealing numerous polygonal to spindle‐shaped cells with a single, irregular nucleus, diffusely distributed to peripheralized chromatin, and occasionally a single distinct, electron‐dense nucleolus (Figure 5). Occasional nuclear membrane invaginations were observed. Cells were closely apposed but lacked desmosomes or clear cell‐to‐cell adhesions. The cytoplasm contained abundant smooth and rough endoplasmic reticulum, with abundant electron‐dense ribosomes arranged variably in typical lamellar formations or less organized, short, lamellar clusters. Granular, electron‐dense secretory granules with two membranes separated by an electron‐lucent rim were occasionally present in the cytoplasm. Granules occasionally contained an electron‐dense core with a surrounding clear matrix halo and a single visible membrane. These structures measured 150–250 nm in diameter. Scattered swollen mitochondria with decreased and disrupted cristae were also found in the cytoplasm. Extracellular spaces contained relatively regularly spaced hexagonal collagen fibrils and other extracellular filaments. The presence of neurosecretory‐type granules, swollen mitochondria, and abundant rough endoplasmic reticulum was consistent with previous reports of neuroendocrine neoplasms, including paragangliomas, and supported the diagnosis of paraganglioma [1, 2, 3, 4].

**FIGURE 5:**
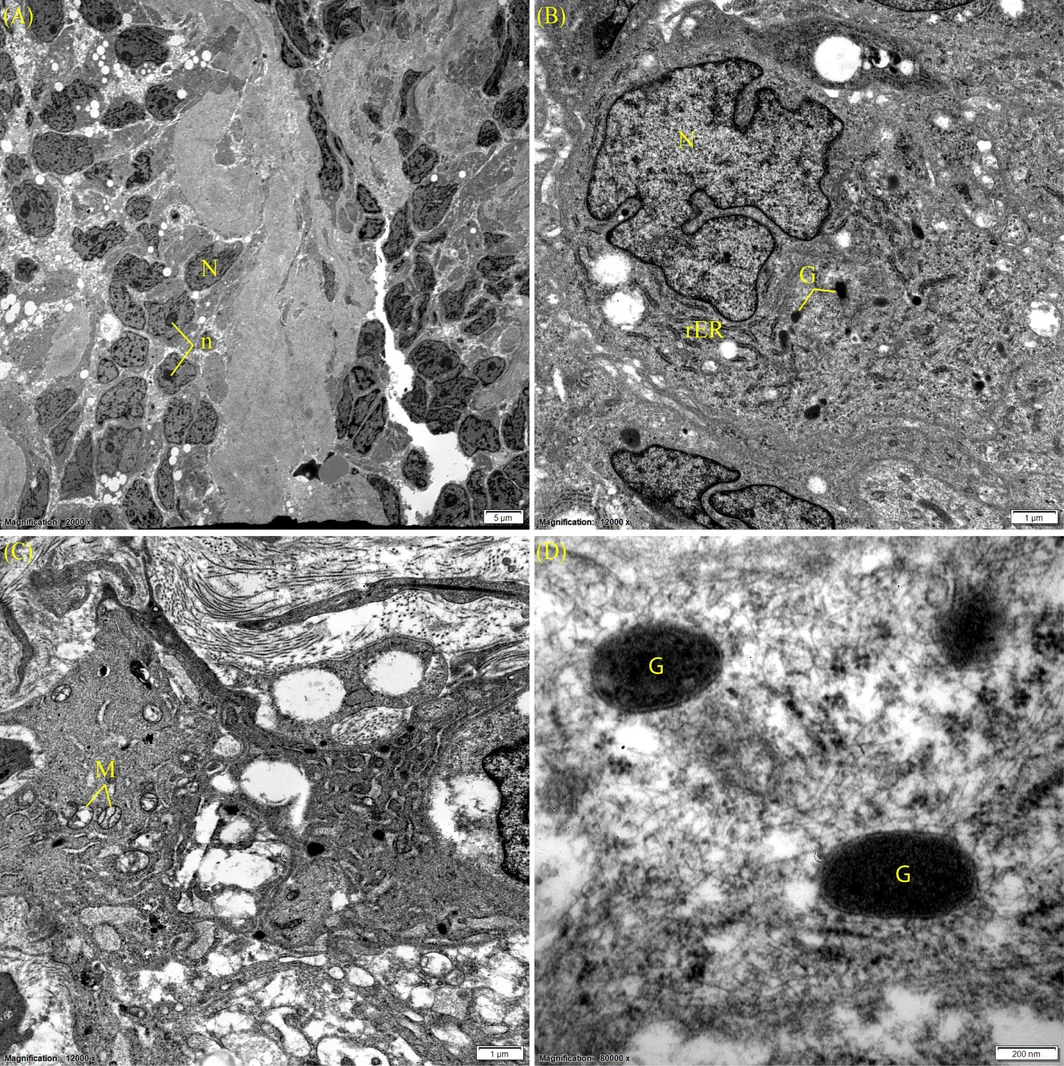
Transmission electron microscopy of the spinal mass obtained through a Tru‐Cut biopsy. (A) Neoplastic cells were polygonal to spindle‐shaped with a single, irregular nucleus (N) and occasionally a single distinct, electron‐dense nucleolus (n). Magnification: 2000×. (B) Cytoplasm contained abundant rough endoplasmic reticulum (rER) and occasional electron‐dense secretory granules (G). Magnification: 12000×. (C) Scattered swollen mitochondria (M) with decreased and disrupted cristae were observed. Magnification: 12000×. (D) Electron‐dense secretory granules (G) were 150–250 nm in diameter with two membranes separated by an electron‐lucent rim. Magnification: 80000×. (A) scale bar = 5 mm. (B, C) scale bar = 1 mm. (D) scale bar = 200 nm.

DNA sequencing was performed to evaluate for the presence of known mutations associated with polycythemia vera in humans and dogs with primary erythrocytosis, and for mutations similar to those found in humans with paragangliomas and erythrocytosis [5, 6, 7, 8, 9, 10, 11, 12, 13, 14, 15, 16, 17, 18, 19]. The patient's genomic DNA was extracted from blood samples, a cheek swab, and the Tru‐Cut biopsy sample of the patient's paraganglioma using the DNeasy Blood & Tissue Kit (Qiagen, 69504). Target regions approximately 400 base pairs in length were amplified via 50 μL PCR reactions using Hot Start Taq 2X Master Mix (NEB, M0496) according to the manufacturer's protocol with primers designed to amplify segments of *JAK2* (V617, C618), *HIF2A* (S71, M368, F374, I412, T519, L529, A530, P531, Y532, I533, P534, M535, D536, G537, D539, F540, L542, P544, Q557, A561), and *PHD1* (61S) and *PHD2* (A228, H374) (Table 1) [5, 6, 7, 8]. Amplicons were verified by agarose gel electrophoresis and purified via DNA Clean & Concentrator columns (Zymo Research, D4014). The purified PCR products were subsequently subjected to Sanger sequencing. Results were compared against sequencing results from blood samples from clinically healthy dogs prepared concurrently in addition to reference genomes (NC_051805.1, NC_051814.1, NC_051808.1). No mutations were identified in any sample based on alignment of the patient's sequencing with the reference genome, as well as with the samples from clinically healthy dogs at the amino acid positions of interest.

**TABLE 1:** Primer sequences used for amplification of gene target regions.

Gene	Targets	Forward primer (5′ → 3′)	Reverse primer (5′ → 3′)	Genome source
JAK2	V617, C618	ATGCATTTCTCTTACAGCAGACAGTATC	GTTTGGGCATTTTAACATATAGCATATT	Adapted from [5]
HIF2A	S71	AGT AGC TCG GAG AGG AGG AA	AAA GGA ATG AAG CAC CGG CAG	NC_051814.1
	M368, F374, I412	GGT GGT CTG AAG CAC CTC TC	TGC TCA GCA CAG TCT GGC CA	NC_051814.1
	T519, L529, A530, P531, Y532, I533, P534, M535, D536, G537, D539, F540, L542, P544, Q557, A561	GGC AGT GCT AGA GCA AGG AAG G	GTG GCA GGG ACA CCT TAT TCC	NC_051814.1
PHD1	S61	ACA CTG CTG CCA TGG ACA GC	ATC CTC TGC CCA CTT CCG TTT G	NC_051805.1
PHD2	A228	GGA GAA GAC GAA CCT GTA C	CAG CTT CCC GTT GCA GTG	NC_051808.1
	H374	CTA TGA ATA CTC CAA CTA CG	ACA TTT AGT ACC AAC TGG G	NC_051808.1

*Note:* PCR thermal cycling conditions: 95°C for 2 min; 28 cycles of 95°C for 20 s, 58°C for 25 s, and 72°C for 1 min; and 72°C for 5 min.

Abbreviations: JAK2, Janus kinase 2; HIF2A, Hypoxia‐inducible factor‐2 alpha; PHD1/2, Prolyl‐hydroxylase domain‐containing protein 1/2.

The dog was treated with palliative radiation (5 Gy × 5 = 25 Gy), gabapentin (100 mg PO q8h), and carprofen (37.5 mg PO q12h). Two weeks post‐treatment, a CBC showed a HCT just above the upper limit of the reference interval (62%; RI 40%–59%). Subsequent CBCs showed a stable HCT within the reference interval (50%–57%) without the need for phlebotomies, and recheck MRIs showed a progressive reduction in the size of the mass. The patient's neurologic deficits completely resolved five months after radiation therapy. Approximately two years following radiation therapy, the patient's clinical signs recurred, including impaired gait as well as fecal and urinary incontinence. At this time, CBCs continued to show a stable HCT within the reference interval (50%–56%). An MRI was performed and showed that the mass had progressed in size. The patient underwent repeat palliative radiation (6 Gy × 5 = 30 Gy) uneventfully and was doing well at home a month later, at the time of manuscript submission, with partial resolution of clinical signs.

## 2 Discussion

Paragangliomas are nonneuronal and nonepithelial neuroendocrine neoplasms derived from neural crest cells [1, 2, 3, 4, 20, 21, 22]. These include paragangliomas that arise from the adrenal medulla (pheochromocytoma), the base of the heart (chemodectoma), the mediastinum, the retroperitoneal space, the tongue, the cauda equina, and the vertebral canal, as in this case. Paragangliomas are rare tumors in dogs, and clinical signs depend on the location and functionality of the neoplasm. Epinephrine‐ or norepinephrine‐secreting paragangliomas can cause weakness, weight loss, anorexia, tachycardia, tachypnea, collapse, panting, hypertension, and exercise intolerance, but some dogs are asymptomatic. In the hospital, this dog had an average heart rate of 123 beats/min (range: 108–156 beats/min) and an average respiratory rate of 31 breaths/min (range: 16–40 breaths/min). Although these values are within the expected range for a small dog in a clinical setting, intermittent tachycardia and tachypnea cannot be excluded. The dog had no other clinical signs attributable to the increased norepinephrine‐creatinine‐ratio, and blood pressure was not measured.

Cytologically, cells from paragangliomas are typically organized in loose cluster‐like arrangements and occasionally form multilayered acinar or rosette‐like arrangements that surround pink, wispy, fibrillar material [20]. Frequent bare nuclei on disrupted cytoplasm are often seen, as expected in neuroendocrine neoplasms, but were not observed in this case. Cells have abundant pale blue cytoplasm and round to oval nuclei with finely stippled chromatin and single or multiple prominent nucleoli. Nuclei have been reported to occasionally have circular, pale‐blue areas that peripheralize chromatin resembling pseudo‐intranuclear inclusions, but were not seen in this case. Paragangliomas may lack features of malignancy, although moderate to marked anisokaryosis and anisocytosis, multiple prominent nucleoli, and multinucleated cells have been reported and were present in this dog.

Histologically, paragangliomas are typically encapsulated, well‐demarcated, lobulated, and densely cellular [1, 20]. Cells are frequently arranged in lobules, nests, bundles, packets, and sheets supported by a fine fibrovascular stroma. Cells have abundant eosinophilic cytoplasm that may appear granular and vacuolated. Nuclei are round and have finely stippled chromatin and 1–3 prominent nucleoli. Positive cytoplasmic immunolabeling for NSE and negative staining for pancytokeratin supported a diagnosis of paraganglioma in this case. The negative immunolabeling with synaptophysin and CgA was atypical, but variable staining patterns have been reported [1, 20, 21, 22]. Canine paragangliomas have also been reported to display positive immunolabeling for vimentin, myelin basic protein, and S‐100, variable immunolabeling for glial fibrillary acidic protein, and negative immunolabeling for PNL2, MelanA, and CD18 [1, 20, 21, 22]. Additional stains were not performed in this case due to no tissues remaining after prioritized tests and stains were performed.

Although histopathology and immunohistochemistry are typically sufficient for the diagnosis of paragangliomas, electron microscopy can provide additional confirmation of neuroendocrine differentiation. Specifically, electron microscopy can identify neurosecretory granules and prominent rough endoplasmic reticulum within neoplastic cells [1]. In this case, electron microscopy identified both findings, which further supported the diagnosis, given the variable immunolabeling observed.

Erythrocytosis refers to an increase in red blood cell mass, measured by an increase in RBC count, hemoglobin, and/or HCT [23]. The term polycythemia is often used synonymously, although it technically refers more broadly to an increase in any hematopoietic cell line. Erythrocytosis can be classified as either relative or absolute. Relative erythrocytosis denotes an increased HCT despite a normal total red blood cell mass, typically resulting from hemoconcentration or splenic contraction. In contrast, absolute erythrocytosis reflects a true increase in total red blood cell mass and can be further subdivided into primary and secondary causes. Primary absolute erythrocytosis is a myeloproliferative syndrome characterized by erythrocyte production that requires little to no EPO. Although not entirely analogous to polycythemia vera in humans, primary erythrocytosis is often referred to as polycythemia vera in dogs. In dogs, primary erythrocytosis usually manifests as isolated erythrocytosis, whereas in humans with polycythemia vera, erythrocytosis is often accompanied by concurrent leukocytosis and thrombocytosis [23]. A JAK2 V617F mutation, analogous to the mutation present in over 90% of human polycythemia vera cases, has been identified in one dog with primary erythrocytosis [5]. Diagnosis of primary erythrocytosis in dogs is a diagnosis of exclusion. Causes of secondary absolute erythrocytosis are categorized as either appropriate or inappropriate. Appropriate secondary erythrocytosis typically arises from increased EPO production secondary to systemic hypoxia and is most often associated with cardiovascular or respiratory diseases. Inappropriate secondary erythrocytosis typically occurs when EPO production is increased in the absence of systemic hypoxia and may result from primary renal disease that results in local hypoxia and thus increased EPO production, EPO‐producing neoplasms, or neoplasms producing substances that stimulate EPO production. Endocrine disorders are also secondary causes of erythrocytosis [23].

There was no evidence of hemoconcentration, respiratory disease, or cardiovascular disease, and no renal cysts or other masses were identified to explain the persistent erythrocytosis in this dog. Primary erythrocytosis is also considered unlikely in this dog, given that DNA sequencing did not identify known associated mutations, and the erythrocytosis resolved after radiation therapy of the spinal mass. EPO production by the neoplasm, reported in several veterinary neoplasms, is also considered unlikely given the low serum EPO [23]. This was not confirmed by performing IHC for EPO due to no remaining tissues. Underlying hypoxia cannot be ruled out, given the lack of arterial blood gas analysis, nor can an endocrinopathy‐associated erythrocytosis be excluded; however, these causes would be unlikely to resolve following radiation therapy for the paraganglioma, and endocrine disorders typically result in only mild erythrocytosis. Finally, a catecholamine‐mediated erythrocytosis cannot be completely dismissed, especially given the elevated urine normetanephrine‐creatinine‐ratio, though this is considered unlikely given the persistent and substantial degree of the erythrocytosis.

Recent reports in the human literature have detailed a syndrome of typically multiple and often metastatic pheochromocytomas/paragangliomas with concurrent erythrocytosis (typically called polycythemia in the literature regarding this syndrome) and elevated EPO, ocular lesions, and occasionally somatostatinomas [6, 7, 8, 9, 10, 11, 12, 13, 14, 15, 16, 17, 18, 19]. This syndrome is termed polycythemia‐paraganglioma syndrome (PPS), polycythemia‐paraganglioma‐somatostatinoma syndrome, or Pacak‐Zhuang syndrome. Most cases are reported in female patients at birth or in childhood, but there are case reports in males as well as in a subset of adult females (termed late‐onset presentation). Prognosis is unclear given the scarcity of reported cases, but the late‐onset cases appear to be milder with no reports of metastatic disease [16]. Treatment consists of surgical resection of tumors when possible, phlebotomies, and medications to help alleviate symptoms caused by the elevation in catecholamines [14].

Most reported cases of PPS in people have been associated with somatic mutations that disrupt regulation of cellular responses to hypoxia involving hypoxia‐inducible factors (HIFs) [6, 7, 8, 9, 10, 11, 12]. HIFs are transcription factors that regulate numerous downstream genes involving erythropoiesis, angiogenesis, autophagy, and energy metabolism [24]. In health under normoxic conditions, HIFs are constantly produced but rapidly hydroxylated by prolyl‐hydroxylases (PHDs) and degraded by the proteasome [9, 10, 24]. Under hypoxic conditions, PHDs are inactivated, and HIFs are translocated to the nucleus, allowing for an appropriate response to low amounts of oxygen [9, 10, 24]. Mutations affecting hypoxia‐inducible factor‐2α (HIF‐2α), prolyl‐hydroxylase 1 (PHD1), and prolyl‐hydroxylase 2 (PHD2) have been demonstrated in the majority of the cases of this syndrome. These mutations prevent the normal degradation of HIF‐2α, leading to its stabilization and continuous activation of hypoxia‐responsive genes in normal oxygen conditions. This persistent activation creates a state of pseudohypoxia, leading to inappropriate erythropoiesis, angiogenesis, and other conditions that promote cellular proliferation, which can result in erythrocytosis and tumorigenesis [6, 7, 8, 9, 10, 11, 12, 13, 14, 15, 16, 17, 18]. Cases in people with similar presentations for which these mutations were not identified are thought to be due to mutations that have not yet been identified. Considering the diversity of reported mutations in humans with PPS, which involve different exons and a variety of missense changes, the absence of identified mutations in this case does not exclude the possibility of a functionally relevant mutation that was not captured by the sequencing approach used [6]. Given the similarities between this dog's presentation and PPS in humans, in addition to the resolution of the HCT following radiation therapy, a somatic mutation resulting in dysregulated cellular responses to oxygen levels could be a contributing factor for the persistent erythrocytosis in this case.

In summary, this dog's paraganglioma decreased in size with radiation therapy, which was associated with improvement of clinical signs and resolution of the erythrocytosis. To our knowledge, this is the first canine case in which parallels with human PPS were explored. Although DNA sequencing did not identify mutations for correlates to mutations found in humans with PPS and the serum EPO levels were low, the concurrent resolution of the erythrocytosis following radiation therapy without additional treatment supports a potential correlation between the paraganglioma and erythrocytosis, perhaps due to an as‐yet unidentified mutation affecting HIFs or PHDs, as has been proposed in PPS in people.

## Conflicts of Interest

The authors declare no conflicts of interest.
